# Trafficking Defect and Proteasomal Degradation Contribute to the Phenotype of a Novel KCNH2 Long QT Syndrome Mutation

**DOI:** 10.1371/journal.pone.0018273

**Published:** 2011-03-31

**Authors:** Anton Mihic, Vijay S. Chauhan, Xiaodong Gao, Gavin Y. Oudit, Robert G. Tsushima

**Affiliations:** 1 Departments of Medicine and Physiology, University of Toronto, Toronto, Ontario, Canada; 2 Division of Cardiology, University Health Network, Toronto, Ontario, Canada; 3 Department of Medicine, Mazankowski Alberta Heart Institute and Division of Cardiology, University of Alberta, Edmonton, Alberta, Canada; 4 Department of Biology, York University, Toronto, Ontario, Canada; Emory University School of Medicine, United States of America

## Abstract

The Kv11.1 (hERG) K^+^ channel plays a fundamental role in cardiac repolarization. Missense mutations in *KCNH2*, the gene encoding Kv11.1, cause long QT syndrome (LQTS) and frequently cause channel trafficking-deficiencies. This study characterized the properties of a novel *KCNH2* mutation discovered in a LQT2 patient resuscitated from a ventricular fibrillation arrest. Proband genotyping was performed by SSCP and DNA sequencing. The electrophysiological and biochemical properties of the mutant channel were investigated after expression in HEK293 cells. The proband manifested a QTc of 554 ms prior to electrolyte normalization. Mutation analysis revealed an autosomal dominant frameshift mutation at proline 1086 (P1086fs+32X; 3256InsG). Co-immunoprecipitation demonstrated that wild-type Kv11.1 and mutant channels coassemble. Western blot showed that the mutation did not produce mature complex-glycosylated Kv11.1 channels and coexpression resulted in reduced channel maturation. Electrophysiological recordings revealed mutant channel peak currents to be similar to untransfected cells. Co-expression of channels in a 1∶1 ratio demonstrated dominant negative suppression of peak Kv11.1 currents. Immunocytochemistry confirmed that mutant channels were not present at the plasma membrane. Mutant channel trafficking rescue was attempted by incubation at reduced temperature or with the pharmacological agents E-4031. These treatments did not significantly increase peak mutant currents or induce the formation of mature complex-glycosylated channels. The proteasomal inhibitor lactacystin increased the protein levels of the mutant channels demonstrating proteasomal degradation, but failed to induce mutant Kv11.1 protein trafficking. Our study demonstrates a novel dominant-negative Kv11.1 mutation, which results in degraded non-functional channels leading to a LQT2 phenotype.

## Introduction

The *KCNH2* gene encodes the Kv11.1 α-subunit (previously referred to as hERG; human ether-à-go-go related gene) of the rapidly activating delayed rectifier K^+^ (I_Kr_) current; the predominant component of cardiac repolarization [1]. To date, over 200 known *KCNH2* mutations have been described causing a variant of long QT syndrome, known as LQT2. The resulting loss of function in I_Kr_ from these mutations can cause syncope or sudden death due to ventricular tachyarrhythmias triggered by early afterdepolarizations [2]–[4]. Standard treatment for patients with LQT2 includes an implantable cardioverter-defibrillator and β-blocker therapy, but there exists a need to develop more tailored treatments as the specific molecular mechanisms underlying LQT2 vary widely.

A predominant cause of Kv11.1 channel dysfunction in LQT2 involves trafficking deficiencies of mutant channels [5]. Single point mutations (missense) in *KCNH2* consistently yield channels characterized by altered or impaired current amplitudes or kinetics [6]. While the vast majority of missense mutations yield nonfunctional channels, some truncated mutants are capable of forming functional channels [7]–[10]. Truncated nonsense mutants can also arise from insertion or deletion mutations producing premature stop codons. In general, these nonsense LQT2 mutants reside at the distal C-terminus, downstream of highly conserved stretches of amino acids including the pore region and domains required for tetramerization, maturation, stability and surface expression of Kv11.1channels [11]–[13]. Interestingly, numerous LQT2 trafficking-deficient mutants can be rescued following specific non-physiologic manipulations of the cell culture conditions [14]. For example, functional rescue has been achieved following 24 h incubation at reduced temperature (∼27°C) [15], incubation with high-affinity pore-blockers (E-4031, cisapride) [15]–[17], proteasomal inhibitors (lactacystin, MG132, ALLN) [18]–[20], lysosome inhibitors (leupeptin; bafilomycin) [18], [20], [21], or aminoglycoside antibiotics (G-418, gentamicin) [10].

In the present study, we characterized a novel LQT2 mutation Kv11.1-P1086fs+32X, causing ventricular fibrillation, which results in dominant-negative suppression of wild-type (wt) Kv11.1 current amplitude. Conventional strategies to rescue channel trafficking were unsuccessful despite the fact that the truncation mutation was located at the distal C-terminus. Incubation of mutant channels with the proteasomal inhibitor lactacystin significantly increased protein expression levels, suggesting that the mechanism underlying dysfunction of this mutant channel involves proteasomal degradation.

## Methods

### Ethics statement

The study protocol conformed to institutional standards and to the Declaration of Helsinki. Written consents were obtained from both patients for clinical testing.

### Clinical evaluation

Patients underwent a physical examination and were assessed for baseline electrolytes as well as evaluated by a 12-lead ECG and echocardiogram assessed using standard criteria. Blood samples were obtained and genomic DNA was extracted then amplified using polymerase chain reaction. Patients were screened for LQTS types 1–5 ion channel mutations using a commercial system (*FAMILION*; PGxHealth, New Haven, CT). Ion channel mutations were confirmed following reamplification, subcloning and restriction digestion. An intravenous epinephrine stress test (0.05 to 0.2 µg/kg/min over 15 minutes) was also performed in the proband's asymptomatic brother as previously described [22].

### DNA constructs

The human Kv11.1 pSP64 DNA was generously provided by Dr. Michael Sanguinetti [23] (University of Utah) and the amino terminal hemagglutinin (HA)-tagged wild-type (wt) Kv11.1 construct was kindly provided by Dr. Alvin Shrier (McGill University, Montreal, QC) [11]. The Kv11.1 frameshift mutation (P1086fs+32X; 3256InsG) was prepared using a Quikchange II site-directed mutagenesis kit (Stratagene, La Jolla, CA). The primers were as follows: Sense: 5′-gct gtg acc acc ccg ggg gcc tgg ccc cac ttc cac atc c- 3′; Antisense: 5′- gga tgt gga agt ggg gcc agg ccc ccg ggg tgg tca cag c-3′. The N-terminal hemaggluttinin (HA)-tagged Kv11.1-wt served as a template. Channel constructs were confirmed by sequencing and the protein was verified with Western blot analysis.

### Cell culture, transfection and drug treatments

HEK293 cells were grown at 37°C in a humidified 5% CO_2_ incubator. Cell culture medium was Dulbecco's Minimum Essential Medium (DMEM) (Invitrogen, Burlington, ON) containing 4.5 g/L glucose and L-glutamine supplemented with 10% fetal bovine serum and penicillin/streptomycin (100 units/mL; 100 µg/mL). HEK293 cells were transiently transfected using Lipofectamine™ 2000 (Invitrogen) according to the manufacturer's instructions. Transient transfection of constructs consisted of 2.0 µg Kv11.1-wt or Kv11.1-mut, 1.0 µg Kv11.1-wt and 1.0 µg Kv11.1-mut or 1.0 µg Kv11.1-wt for the equivalent of a 35 mm dish. A Kv11.1-wt construct possessing a HA-tag (Kv11.1-HA-wt) was used in some experiments. For electrophysiology experiments, cells were also co-transfected with green fluorescence protein (GFP) (0.25 µg) to visualize positively transfected cells, and 24 h after transfection, cells were trypsinized and placed in 35 mm dishes in low density and cultured overnight for single-cell electrophysiological recordings. For biochemical experiments, cells were grown in 100 mm dishes and the amount of DNA used was 6 times that mentioned above. Twenty-four hours after transfection, some cells were incubated at 30°C incubator or with specific drugs for 24 h before use in electrophysiological or molecular biology experiments. These drugs included 5 µM E-4031 (Calbiochem, San Diego, CA) and 20 µM lactacystin (Sigma-Aldrich Canada, Oakville, ON) that were prepared as stock solutions dissolved in water. Cells were washed and incubated in drug-free medium for 1 h before electrophysiological experiments.

### Electrophysiology

Voltage-gated K^+^ channel (Kv11.1) recordings of single cells were performed using the whole-cell configuration of the patch clamp technique. Recordings were acquired using a HEKA EPC-10 amplifier operating at a sampling rate of 2.5 kHz and low-pass filtered at 2.0 kHz and recorded with Pulse software (HEKA Electronics Inc, Mahone Bay, NS). Pipettes were pulled from 1.5 mm borosilicate glass capillary tubes (World Precision Instruments, Sarasota, FL) using a programmable micropipette puller (Sutter Instrument, Novato, CA). Pipettes were heat polished and resistances were obtained ranging from 2–4 MΩ when filled with a solution containing (in mM): 140 KCl, 1 MgCl_2_, 5 EGTA, 10 HEPES, 5 MgATP (pH 7.2 with KOH). The bath solution contained (in mM): 140 NaCl, 4 KCl, 1 CaCl_2_, 1 MgCl_2_, 10 glucose, and 5 HEPES (pH 7.4 adjusted with NaOH). Once whole-cell configuration was established, cells were held at −80 mV and subjected to various experimental protocols. All recordings were performed at room temperature (∼22°C). No leak subtraction was used during current recording and current densities were normalized to cell capacitances.

Kv11.1 gating kinetic analysis was performed as previously described by Zhou and colleagues [24]. Briefly, Kv11.1 activation time constants were obtained by fitting the rising phase of continuous currents following 3 s depolarizing pulses from −30 to 0 mV with a mono exponential function. At test potentials greater than 0 mV, time constants were obtained by fitting families of envelope tail currents produced at various voltages (20, 40 and 60 mV) because of the influence of channel inactivation. Deactivation kinetics were measured from tail currents (−120 to −20 mV) following a prepulse depolarization to +60 mV. Decaying tail currents were fit with a double exponential function. Steady-state inactivation was assessed using a triple-pulse protocol consisting of a 2-s depolarizing pulse to +60 mV followed by step repolarizations from −140 to +20 mV. Data were fit with a Boltzmann function. The time constants for Kv11.1 fast inactivation were obtained using the triple-pulse protocol function. A prepulse depolarization of +60 mV was followed by a rapid −100 mV repolarizing pulse and subsequent step depolarizations −20 to +60 mV were fit with a mono exponential function. Time constants for recovery from inactivation were obtained using a brief depolarizing pulse to +60 mV followed by repolarizing pulses from −100 to −20 mV. The resulting currents were fit with a mono exponential function.

### Western blot

HEK293 cells were grown to similar confluences and transfected with the indicated cDNA constructs. Lysis buffer contained 0.5% Nonidet P-40 buffer; 50 mM Tris HCl (pH = 8.0); 75 mM NaCl and protease inhibitor cocktail (Roche Diagnostics Canada, Laval, QC). Lysates were cleared of DNA and cellular debris, protein concentrations were determined and equal amounts of lysate (20 µg/lane) were subjected to SDS-polyacrylamide gel electrophoresis (7.5% gel) followed by transfer onto PVDF membranes. After blocking, membranes were incubated with primary antibodies directed against the Kv11.1 C-terminus (APC-016, 1∶2000; Alomone, Jerusalem, Israel) or against the HA-epitope (H-9658, 1∶5000; Sigma) and secondary antibodies were horseradish peroxidase-conjugated (Jackson Immunoresearch, West Grove, PA). Signals were detected using ECL plus (GE Healthcare, Baie d'Urfe, QC) and exposed to X-ray films, which were developed using an automated photo-processor device.

### Co-immunoprecipitation

HEK293 cells grown on 100 mm plates were transfected with Kv11.1-wt and Kv11.1-mut constructs and harvested after 48 h incubation. Cells were lysed with buffer containing 1% Triton X-100, 50 mM Tris-HCl pH 8.0, 150 mM NaCl, 1 mM CaCl_2_, and a protease inhibitor cocktail (Roche). Lysates were precleared with a 50% slurry of Protein A Sepharose CL-4B resin (GE Healthcare) and supernants were subsequently retained for incubation with Protein A beads and 2 µL of antibody. Reciprocal co-immunoprecipitation was performed with anti-Kv11.1-wt antibody (epitope corresponding to C-terminal 16 amino acids) or anti-HA antibody for recognition of the mutant Kv11.1 channels. After binding overnight at 4°C, complexes were washed 3–4 times with wash buffer containing 0.1% Triton X-100, 10 mM Tris-HCl pH 8.0, and 150 mM NaCl. Boiling the samples prior to SDS-PAGE eluted proteins and membranes were immunoblotted for the putative interacting protein partner.

### Immunocytochemistry and confocal microscopy

HEK293 cells were transfected with Kv11.1-wt and Kv11.1-mut constructs as well as GFP (0.25 µg) and were subsequently re-plated onto glass coverslips 24 h after transfection. After an additional 12–24 h, cells were fixed with 2% paraformaldehyde. For total Kv11.1 protein expression, cells were permeabilized with 0.5% Triton X-100 and probed with an anti-HA antibody (Sigma, H-2095), while total surface membrane Kv11.1 was probed in non-permeabilized cells with an anti-Kv11.1 antibody (Alomone, APC-109) that recognizes an extracellular 16-amino acid epitope located between S1 and S2. The antibody was tested and determined to have roughly equivalent binding affinity to both Kv11.1-wt and Kv11.1-mut constructs. Cells were blocked with 5% goat serum, incubated with primary antibodies for 2 hours at room temperature and then incubated with CY3-conjugated secondary antibodies for 1 hour at room temperature. Cells were also incubated with Alexa Fluor 633-phalloidin (1∶40) for 20 minutes at room temperature and DAPI (1∶2000) for 3 minutes at room temperature. Imaging was performed on a 4-channel Olympus FV1000 laser scanning confocal microscope with a 60× oil immersion objective, and with diode laser excitations at 405 nm for DAPI, 473 nm for GFP, 559 nm for Alexa 568 and 635 nm for Alexa 633. Untransfected cells, and cells transfected with only GFP served as negative controls. All groups within each experiment were imaged with the same signal amplitude, gain and laser intensity. Images are of representative cells for each transfection condition.

### Data analysis and statistics

Electrophysiological measurements were analyzed using Microcal Origin v6.0 (OriginLab Corporation, Northampton, MA). Digital images of molecular biology experiments were quantified using Image J (NIH, Bethesda, MD). Quantification of immunoblot band optical density was calculated by determining the sum of the pixel intensity for each band subtracted by the average background. Line scan analyses of single-slice confocal images were obtained using Olympus Flouview Version 1.7 software (Olympus Corporation, Markham, ON). For all figures, data points represent mean ± S.E.M. and “n” is the number of experiments per group. Origin was used to analyze data and fit exponential functions to raw current traces. An unpaired Student's t test was used to compare control values to experimental groups. In experiments comparing more than 2 groups, one-way analysis of variance (ANOVA) for repeated measures was calculated and followed by Bonferroni's post hoc test to assess differences between groups. A p<0.05 was considered statistically significant.

## Results

### Patient Characteristics and Mutation Analysis

**Figure 1:**
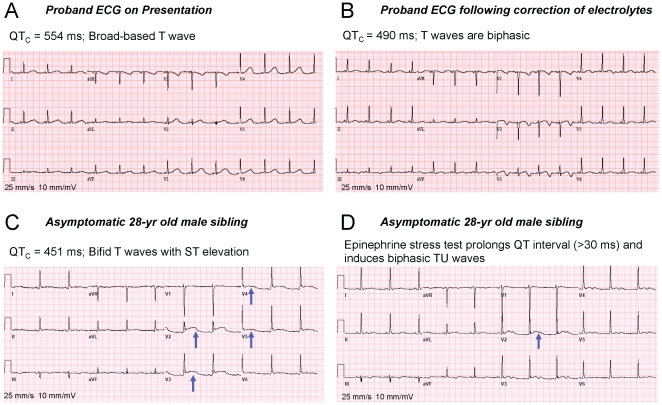
12-lead ECG of proband and sibling. QTc prolongation was observed before (A) and after (B) electrolyte restoration. C: The asymptomatic sibling carried the same LQT2 mutation and ECG demonstrated mild QTc prolongation with biphasic T waves. D: Epinephrine stress testing was consistent with definite LQTS. Blue arrows denote ST elevation.

The proband was a 33-year old female with no prior cardiac history, who suffered a ventricular fibrillation arrest following a minor automobile accident. There was no family history of sudden death or syncope. Her serum electrolytes on presentation were significant for severe hypokalemia and hypomagnesemia (K^+^ = 2.7 mM; Mg^2+^ = 0.63 mM; and Ca^2+^ = 2.1 mM). Her baseline 12-lead ECG showed marked QTc prolongation (554 ms) with a broad-based T wave that was not related to medications (Figure 1A). Echocardiogram showed no structural heart disease with normal biventricular size and function. Following correction of her electrolyte abnormalities, her QTc remained prolonged (490 ms) and her T waves became biphasic (Figure 1B). The proband was diagnosed with LQTS and treated with an implantable cardioverter-defibrillator along with oral Mg^2+^ and K^+^ supplementation and β-blocker therapy. The proband's 28-year-old brother was asymptomatic, but manifested mild QTc prolongation (451 ms) with bifid T waves and resting ST segment elevation (Figure 1C). Following epinephrine stress testing, his uncorrected QT interval prolonged by >30 ms accompanied by the development of biphasic T waves and prominent U waves, consistent with LQTS (Figure 1D). Therefore, he was placed on prophylactic β-blocker therapy.

**Figure 2:**
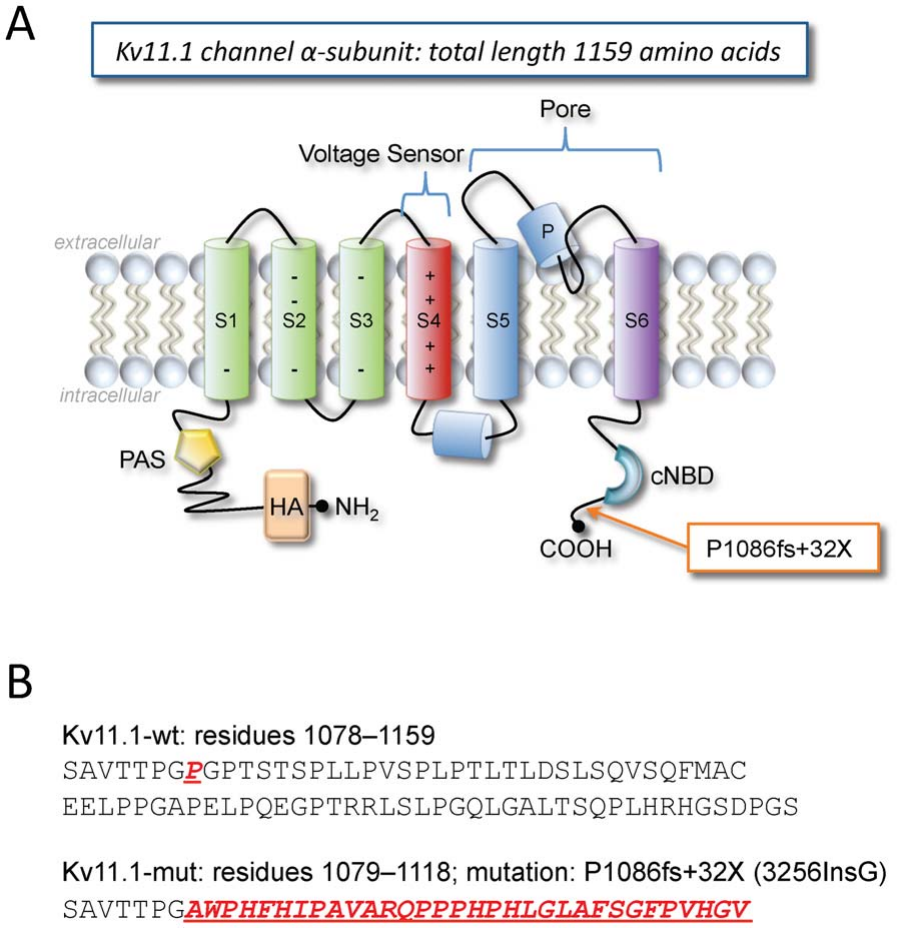
Kv11.1 α-subunit schematic and sequence of channel mutation. A: Located at the C-terminus, the P1086fs+32X (3256InsG) mutation is caused by a guanosine insertion in the codon at position 3256 (2356InsG), which elicits a frameshift at proline 1086 and produces 32 new amino acids before a premature stop codon. The mutation is downstream of the cyclic nucleotide binding domain (cNBD) and produces a truncated channel subunit. The N-terminus contains the Per Arnt Sim domain (PAS) and a HA-tag. B: Sequences for Kv11.1-wt and Kv11.1-mut (P1086fs+32X) including the nonsense 32 amino acid sequence.

Sequence analysis revealed that the proband and her brother both possessed a point mutation in *KCNH2*. A guanosine insertion in the codon sequence at nucleotide 3256 produced the frameshift mutation localized at the Kv11.1 channel C-terminus (P1086fs+32X; Figure 2). As a result, a premature stop codon occurred downstream of 32 nonsense amino acids producing a truncated 1118 amino acid Kv11.1 channel α-subunit. This point mutation is located downstream of the cyclic nucleotide binding domain (residues 750–870) [25] and the R-X-R ER-retention signal sequence (1005–1007) [26], but overlaps with the tetramerizing coiled-coil domain (residues 1018–1122) [13].

### Properties of Kv11.1 P1086fs+32X channels

**Figure 3:**
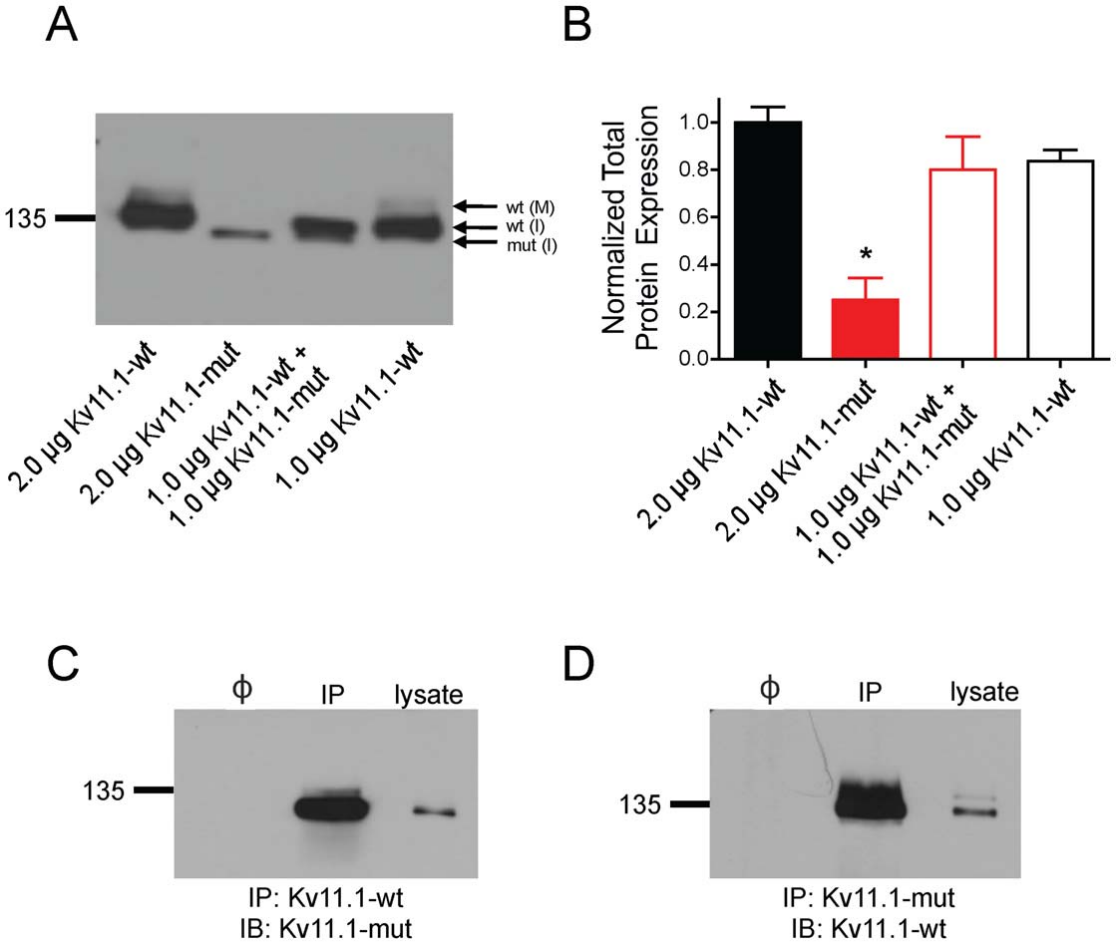
Biochemical analysis Kv11.1 protein expression and binding. A: Immunoblot of equal amounts of protein lysates (25 µg) from HEK cells transfected with 1.0 or 2.0 µg of Kv11.1 cDNA. Kv11.1-wt channels expressed two protein bands corresponding to an immature core-glycosylated 135 kDa ER-resident Kv11.1 protein [wt-(I)], and a mature complex-glycosylated 155 kDa Kv11.1 band [wt-(M)]. Mutant Kv11.1 channels produced a single band at a slightly lower molecular weight (predicted to be 4 kDa smaller than Kv11.1-wt, thus approximately 131 kDa) corresponding to an immature core-glycosylated Kv11.1-mut protein [mut-(I)]. B: Densitometric analysis of total Kv11.1 protein (n = 4 experiments) demonstrated that Kv11.1-mut transfections resulted in significantly less total Kv11.1 protein expression than control or co-transfection (ANOVA *p<0.01). C,D: Reciprocal co-immunoprecipitation of Kv11.1-wt and Kv11.1-mut channels. Cells were transfected with a Kv11.1-wt construct lacking the HA-tag and Kv11.1-HA-mut. Co-immunoprecipitation was performed with anti-Kv11.1-wt antibody (C) (epitope corresponding to C-terminal 16 amino acids) or anti-HA antibody (D) for recognition of Kv11.1-mut. The two channel constructs strongly interacted. (Φ is a sample in which primary antibody was excluded during binding; IP: immunoprecipitation; IB: immunoblot).

To assess the properties of the Kv11.1-mut channels, Western blotting was performed and total Kv11.1 protein expression was quantified (Figure 3A and B). HA-tagged constructs allowed for the simultaneous detection of full-length Kv11.1-wt and Kv11.1-mut channel protein. We observed a similar protein expression level when both 2.0 µg and 1.0 µg of Kv11.1-wt construct was transfected in 35 mm dishes. The total Kv11.1 protein expression was normalized to 2.0 µg Kv11.1-wt control (n = 4 experiments per group). Total Kv11.1 protein expression for 1.0 µg Kv11.1-wt alone and 1.0 µg Kv11.1-wt+1.0 µg Kv11.1-mut was not different from control (0.84±0.05 and 0.80±0.14, respectively). However, the total Kv11.1 protein expression level for 2.0 µg Kv11.1-mut was significantly reduced compared to Kv11.1-wt control (0.25±0.09; p<0.01) and there was a complete absence of the corresponding mature complex-glycosylated Kv11.1 band. The KV11.1-wt 155 kDa complex glycosylated band was noticeably absent in the co-transfection group despite roughly normal total protein expression levels.

To examine the possibility of heterotetrameric channel formation following coexpression, we performed reciprocal co-immunoprecipitation on Kv11.1-wt and Kv11.1-HA-mut channels (Figure 3C and D). This was achieved by utilizing a non-HA-tagged Kv11.1-wt construct and a Kv11.1 antibody corresponding to a 16 amino acid epitope located at the distal C-terminus of the wild-type channel. Alternatively, HA-tagged Kv11.1-mut was immunoblotted using the standard anti-HA antibody. The channels strongly interacted; however, we could not detect the presence of a mature complex-glycosylated protein band at 155 kDa, indicating that heterotetrameric channels do not undergo normal protein maturation.

**Figure 4:**
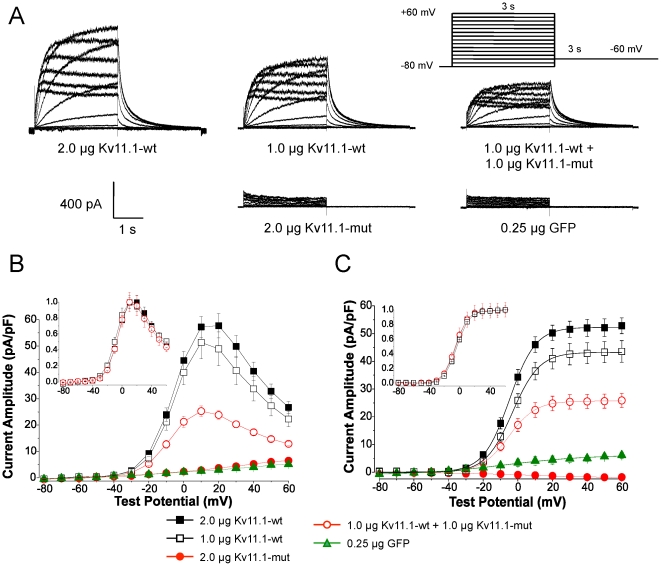
Kv11.1 P1086fs+32X mutation reduces peak current amplitude following coexpression with Kv11.1-wt. Electrophysiological properties of Kv11.1-wt and Kv11.1-mut channels were assessed using whole-cell patch clamping. A: Families of current tracings from −80 to +60 mV following 3 s step depolarizations. Kv11.1-wt currents were reduced following coexpression with Kv11.1-mut, indicating a dominant-negative suppression currents. Kv11.1-mut constructs were indistinguishable from GFP-transfected controls. B: The current-voltage relationship demonstrated that peak current amplitude is significantly reduced following coexpression (2.0 µg Kv11.1-wt, 57.7±4.6 pA/pF, n = 16; 1.0 µg Kv11.1-wt, 51.4±6.3 pA/pF, n = 14; 1.0 µg Kv11.1-wt+1.0 µg Kv11.1-mut, 25.3±2.0 pA/pF, n = 10, p<0.001 from Kv11.1-wt). Peak Kv11.1-mut currents were similar to GFP-transfected cells (2.0 µg Kv11.1-mut, 6.5±0.8 pA/pF, n = 15 versus 0.25 µg GFP, 5.1±0.5 pA/pF, n = 5). The current-voltage profile and C-type inactivation properties were identical following normalization (inset). C: Peak tail currents were measured immediately following repolarization. Kv11.1-wt+Kv11.1-mut tails were significantly reduced compared to control (2.0 µg Kv11.1-wt, 52.8±2.8 pA/pF, n = 16; 1.0 µg Kv11.1-wt, 43.5±3.9 pA/pF, n = 14; 1.0 Kv11.1-wt+1.0 µg Kv11.1-mut, 25.9±2.6 pA/pF, n = 10; p<0.01 from Kv11.1-wt). Tail currents were normalized and fit to a Boltzmann function to assess the steady-state activation properties (inset). No changes in slope or V1/2 parameters were observed.

Heterologous expression with Kv11.1-wt and Kv11.1-mut in a 1∶1 ratio resulted in a suppression of Kv11.1 current amplitude at all voltages positive to −20 mV, indicating a dominant-negative suppression of wild-type current (Figure 4A). The current-voltage relationship revealed that peak current amplitudes at +20 mV were significantly reduced following coexpression of Kv11.1-wt and Kv11.1-mut constructs (Figure 4B). Following normalization, these three groups possessed identical current-voltage profiles and normal C-type inactivation (Figure 4B, inset). Kv11.1-mut alone did not produce measurable currents and were not different from cells transfected with GFP alone.

Peak tail current amplitudes measured at −60 mV immediately following a +60 mV pulse, were also reduced following coexpression of Kv11.1-wt and Kv11.1-mut constructs (Figure 4C). Individual tail currents for each group were normalized and fit to a Boltzmann function to determine the voltage-dependence of current activation and produce a steady-state activation curve (Figure 4C, inset). There was no difference in slope or V_1/2_ parameters between these groups (Table 1). Additionally, Kv11.1-mut constructs alone did not produce any measurable tail currents, suggesting that mutant constructs did not form functional channels.

**Table 1:** Summary of steady-state channel kinetics.

Steady-state activation characteristics			
	*Midpoint of current activation V_½_ (mV):*	*Slope factor:*	*n-value*
K_V_11.1-wt	−3.18±0.85	7.60±0.36	10
K_V_11.1-wt+K_V_11.1-mut	−4.35±1.09	7.59±0.21	14

Steady-state inactivation characteristics			
	*Midpoint of current inactivation V_½_ (mV):*	*Slope factor:*	*n-value*
K_V_11.1-wt	−45.15±2.47	20.6±1.87	6
K_V_11.1-wt+K_V_11.1-mut	−46.8±1.71	20.1±0.65	8

**Figure 5:**
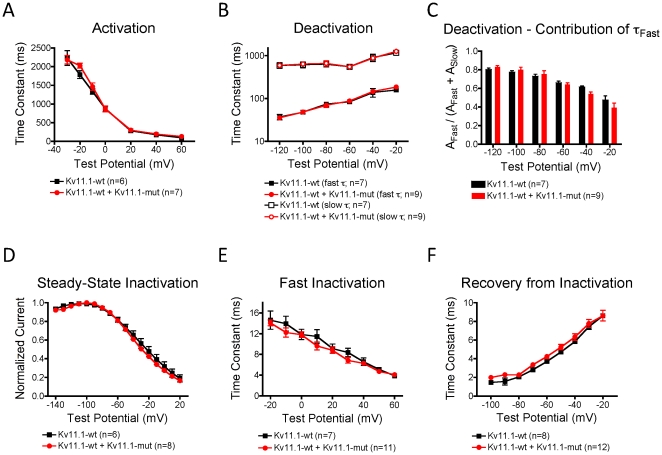
Detailed analysis of Kv11.1 kinetics. Channel kinetics were compared between Kv11.1-wt and Kv11.1-wt+Kv11.1-mut groups as no appreciable currents could be measured from Kv11.1-mut alone. There was no difference in channel activation (A), deactivation (B), contribution of the fast component to current decay (C), steady-state inactivation (D), fast inactivation (E) or recovery from inactivation (F).

We performed an exhaustive assessment of Kv11.1 channel kinetics but observed no differences in the rates of activation, deactivation, recovery, fast inactivation and steady-state inactivation parameters following coexpression of Kv11.1-wt and Kv11.1-mut channel constructs (Figure 5 and Table 1). This evidence strongly suggested that P1086fs+32X affects the total number of functional Kv11.1 channels present at the plasma membrane, and most likely does not alter the open probability of channel opening.

**Figure 6:**
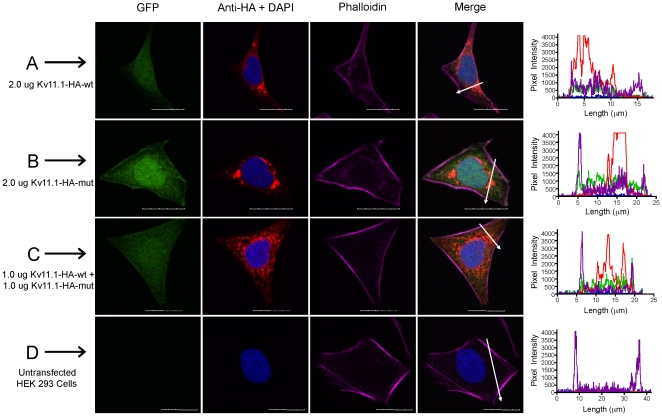
Total Kv11.1 protein expression in fixed, permeabilized cells. The staining patterns for cells co-transfected with GFP (green) and HA-tagged Kv11.1 plasmids (CY3, red) were assessed using immunocytochemistry and confocal microscopy. A: Kv11.1-wt; B: Kv11.1-mut; C: co-expression of both plasmids. Untransfected cells served as negative controls (D). DAPI stained nuclei (blue) and phalloidin stained actin filaments (CY5, purple) were used to identify the nucleus and plasma membrane, respectively. White arrows indicate the location of line scans through the plasma membrane and perinuclear regions of merged images. Profile histograms indicate the fluorescence intensity for pixels along line scans for each group. Scale bar represents 20 µm.

Total Kv11.1 protein cellular localization and expression was assessed by immunocytochemistry and confocal microscopy (Figure 6). Single plane XY confocal scans were taken through cells, and line scans through the plasma membrane and perinuclear regions were used to demonstrate differences in the expression patterns of Kv11.1 channels at these distinct intracellular locations. Kv11.1-wt protein was detected throughout the cytoplasm and at the plasma membrane and was in contrast to Kv11.1-mut cells, which had dense punctate intracellular protein expression with periplasmic distribution. Co-expression of both plasmids resulted in a hybrid staining phenotype with punctate staining throughout the cytoplasm and less plasma membrane expression.

**Figure 7:**
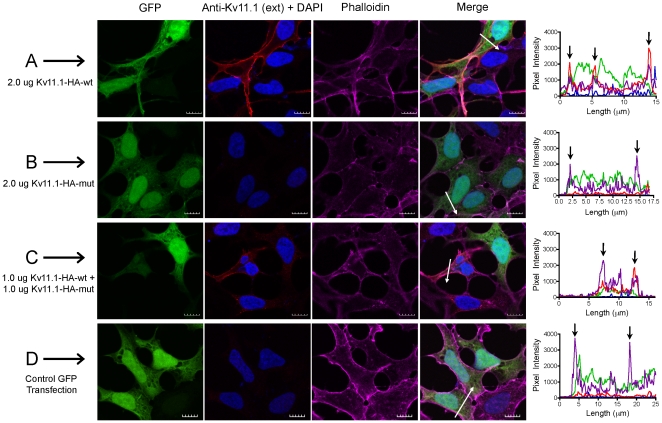
Membrane Kv11.1 protein expression in fixed non-permeabilized cells. Mature Kv11.1 protein expression was investigated using an external Kv11.1 epitope (CY3, red). A: Kv11.1-wt; B: Kv11.1-mut; C: co-expression of Kv11.1-wt and Kv11.1-mut. GFP-transfected cells served as negative controls (D); DAPI stained nuclei (blue); phalloidin stained actin filaments (CY5, purple). White arrows indicate the location of line scans through the plasma membrane and perinuclear regions of merged images. Profile histograms indicate the fluorescence intensity for pixels along line scans for each group. Black arrows indicate the approximate location of plasma membrane in the histogram panels. Scale bar represents 10 µm.

Specific surface expression of Kv11.1 channels was determined by probing non-permeabilized cells using an anti-Kv11.1 antibody that recognizes an external epitope located between the S1 and S2 transmembrane domains of the channel (Figure 7). Kv11.1-wt cells possessed robust expression of Kv11.1 protein at the plasma membrane, as exemplified by the line scan histogram of the merged image. Kv11.1 expression was not detectable in Kv11.1-mut cells. Coexpression of Kv11.1-wt and Kv11.1-mut yielded reduced plasma membrane Kv11.1 expression and areas of punctate staining at the membrane. GFP co-transfection was used as a negative vehicle control for cells not transfected with a channel construct (Figure 7D).

### Mechanism of P1086fs+32X dominant-negative suppression of Kv11.1 channel function

**Figure 8:**
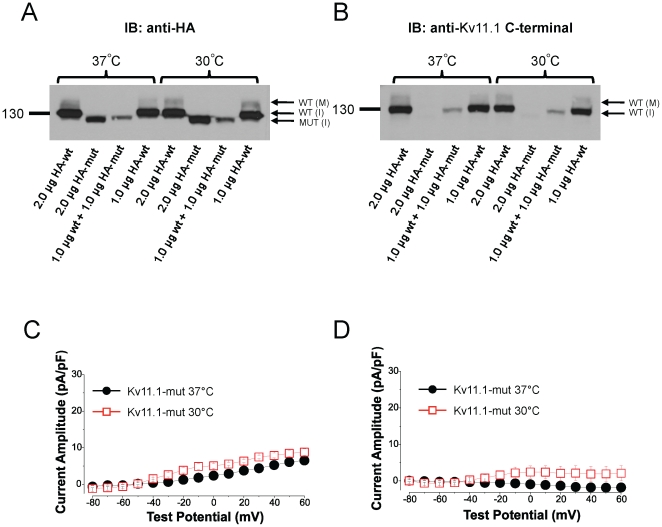
Reduced temperature does not rescue Kv11.1-mut trafficking. A/B: Cells were incubated at 30°C for 24 h and total Kv11.1 protein was assessed by Western blot. Reduced temperature did not change the intensity of the protein band nor cause the appearance of a Kv11.1-mut mature protein band. Co-transfection of non-HA-tagged Kv11.1-wt and HA-Kv11.1-mut (1.0 µg wt+1.0 µg HA-mut; in lanes 3 and 7) allowed for the specific identification of Kv11.1-mut protein (A; anti-HA antibody) and Kv11.1-wt protein (B; anti-Kv11.1 C-terminal antibody). C: Peak current-voltage relationship for Kv11.1-mut alone at 37°C and 30°C revealed no change in current density (Kv11.1-mut at 37°C, 6.5±0.8 pA/pF, n = 15 versus Kv11.1-mut at 30°C, 8.8±0.9 pA/pF, n = 4). D: Peak tail current amplitude did not significantly change with reduced temperature (Kv11.1-mut at 37°C, −1.8±0.3 pA/pF, n = 15 versus Kv11.1-mut at 30°C, 2.1±2.0 pA/pF).

Numerous trafficking-deficient LQT2 mutants can produce functional channels following incubation at reduced temperature. We tested whether P1086fs+32X+Kv11.1-wt trafficking could be rescued following 24 h incubation at 30°C (Figure 8A and B), which yields the highest expression levels of functionally active mature Kv11.1 channels *in vitro* [27]. We compared immunoblots of transiently transfected cells at 37°C and 30°C using anti-HA and anti-Kv11.1 (C-terminal epitope) antibodies. Reduced temperature had no effect on total Kv11.1 protein signal in any of the groups, and more critically, it did not increase the expression nor yield a mature 155 kDa Kv11.1 band following coexpression of 1.0 µg Kv11.1-wt (no HA-tag)+1.0 µg Kv11.1-HA-mut channels. In support of these biochemical assays, whole-cell electrophysiological measurements did not substantially increase peak Kv11.1 currents at +60 mV (Figure 8C). Similarly, peak tail currents measured at −60 mV following the +60 mV step were not significantly enhanced following reduced temperature incubation (Figure 8D).

**Figure 9:**
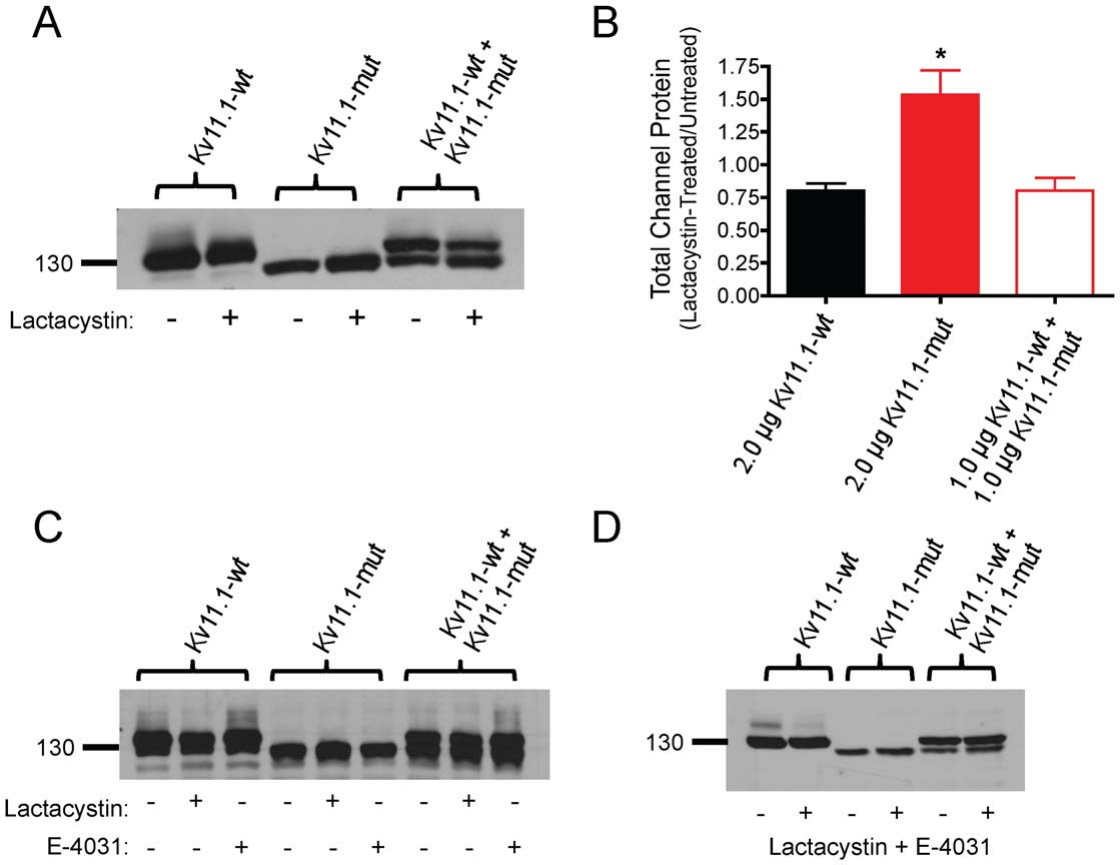
Trafficking of Kv11.1-mut channels cannot be rescued to the plasma membrane. A: Incubation with the proteasomal inhibitor lactacystin (20 µM) for 24 h enhanced the expression of immature Kv11.1-mut protein, but did produce a complex-glycosylated Kv11.1-mut protein. B: Densitometric analysis of total protein expression after lactacystin treatment (+) normalized to non-treated lysates (−). There was a significant increase in the expression of total Kv11.1-mut protein compared to the other groups (ANOVA *p<0.01). Untreated Kv11.1-mut cells (2.0 µg Kv11.1-mut, 1.53±0.19, n = 5) versus 2.0 µg Kv11.1-wt control (0.80±0.05) and 1.0 ug Kv11.1-wt+1.0 µg Kv11.1-mut (0.80±0.10, n = 3). C: Twenty-four h treatment with the Kv11.1 channel blocker E-4031 (5 µM) enhanced the mature Kv11.1 protein band in Kv11.1-wt and Kv11.1-wt+Kv11.1-mut groups, but did not elicit a mature Kv11.1-mut channel. D: Combined 24 h treatment with lactacystin (20 µM) and E-4031 (5 µM) did not significantly enhance Kv11.1-mut protein expression, nor did it rescue channel maturation in the Kv11.1-mut or Kv11.1-wt+Kv11.1-mut groups.

We hypothesized that the P1086fs+32X Kv11.1 mutation results in proteasomal degradation, thereby preventing both homotetrameric mutant channels, and heterotetrameric channels from undergoing the normal maturation process including complex glycosylation and cell-surface expression. Therefore, we tested the specific proteasomal inhibitor lactacystin (20 µM, 24 h) before harvesting cells for Western blot analysis (Figure 9A and B). Lactacystin treatment significantly increased the ratio of total Kv11.1-mut protein normalized to untreated Kv11.1-mut cells versus 2.0 µg Kv11.1-wt control and 1.0 ug Kv11.1-wt+1.0 µg Kv11.1-mut normalized to their untreated controls. Finally, electrophysiological recordings showed no change in peak Kv11.1-mut currents at +20 mV following treatment with lactacystin (2.0 µg Kv11.1-mut, 6.5±0.8 pA/pF, n = 15 versus 2.0 µg Kv11.1-mut+lactacystin, 6.8±0.7 pA/pF, n = 3).

We further hypothesized that combination treatment of Kv11.1-mut channels with proteasomal inhibition and reduced temperature may enhance channel maturation and trafficking. This combination did not significantly alter total Kv11.1 protein expression, nor induce the appearance of a mature Kv11.1 protein band by Western blot (not shown). We alternatively tested the high-affinity Kv11.1 channel blocker E-4031, which has also been shown to rescue trafficking-deficient LQT2 mutants. Twenty-four hour treatment with 5 µM E-4031 alone enhanced the expression of the 155 kDa mature Kv11.1 band in the Kv11.1-wt and Kv11.1-wt+Kv11.1-mut groups, but did not affect the expression Kv11.1-mut control (Figure 9C). Additionally, combination treatment with 5 µM E-4031 and 20 µM lactacystin had no significant effect on Kv11.1-mut protein expression and did not promote the appearance of a mature Kv11.1 protein band in the Kv11.1-mut group (Figure 9D).

## Discussion

### Main Findings

This study investigated the biophysical properties and molecular characteristics of a novel LQT2 mutation P1086fs+32X that resulted in dysfunctional Kv11.1 channels and a clinical phenotype in our patient. Although the mutation is located at the distal C-terminus, it does not undergo normal channel maturation or trafficking to the plasma membrane. In coexpression systems, and most likely in our LQT2 patients, the mutation suppresses wild-type Kv11.1 currents in a dominant-negative fashion. The mutant protein is degraded and cannot be rescued with previously described *in vitro* methods, including incubation at reduced temperature, medium supplemented with high-affinity channel blocker, proteasome inhibitor, or combination of these treatments.

### ER-retention sequence and proteasomal degradation

A number of C-terminal truncation and frameshift LQT2 mutants have been characterized and shown to produce functional Kv11.1 channels when expressed alone [10], [17], [28], [29]. Furthermore, numerous trafficking-deficient LQT2 mutants with abnormalities of the C-terminus can be rescued by utilizing the aforementioned conditions. Therefore, we did not anticipate that the P1086fs+32X LQT2 mutant would have such a profound inhibitory effect on wild-type Kv11.1 channels. This mutation occurs in the Kv11.1 channel tetramerizing coiled-coil domain and is in close proximity to the R-X-R ER-retention sequence [13], [26]. It is therefore conceivable that this mutant channel could interrupt normal channel folding including tetramerization and maturation. Reciprocal co-immunoprecipitation demonstrated that wild-type and P1086fs+32X channels interact, suggesting that heteromeric proteins form. But the mutant channels undergo proteasomal degradation; a process partially inhibited by lactacystin treatment. Therefore, it is likely that the location of the truncation and the addition of subsequent nonsense amino acids may serve to expose the R-X-R ER-retention sequence, thereby marking the mutant protein for proteasomal degradation [19]. The proteasomal protein degradation pathway has emerged as an important mechanism of control of protein levels and function and this study highlights the importance of the proteasomal degradation of I_Kr_ as a key determinant of the function of these ion channels [30], [31].

### Pharmacological chaperones may enhance heteromeric channel maturation

Homomeric Kv11.1-mut channels did not produce an additional Western blot band corresponding to mature channel protein following E-4031 treatment, nor did they elicit functional current. However, the dominant-negative inhibition of Kv11.1-wt+Kv11.1-mut heteromeric channels was partially attenuated by E-4031. Under control conditions, the production of heteromeric proteins yield the phenotype of mutant subunits, which are recognized by the intrinsic quality control components of the ER-Golgi network, targeting them for degradation [32]. In the presence of E-4031, Kv11.1-wt+Kv11.1-mut channel trafficking may have been enhanced due to a pharmacological chaperone mechanism that is believed to drive enhanced channel maturation efficiency [16]. Pharmacological chaperones may serve to disrupt the interaction of immature Kv11.1 proteins with ER chaperone and quality-control proteins including Hsp40 (40-kDa heat shock protein), Hsc70 (70-kDa heat shock cognate protein), Hsp90 (90-kDa heat shock protein), FLBP38, calnexin, and numerous other chaperones, thereby protecting mutant proteins from degradation and alternatively by attenuating digestion by enzymes such as trypsin [33]–[35]. Although this high dose pharmacological approach is not useful therapeutically, it does serve to illustrate a key feature related to the mechanism of Kv11.1 P1086fs+32X channel dysfunction in our patients that possess both normal and mutant alleles [36]. Therefore the heteromeric model is a more accurate representation of the true pathophysiology in the clinical setting.

### Implications for treatment of LQT2 syndrome

An interesting clinical observation in our study was that the proband presented with severe hypokalemia at the time of ventricular fibrillation arrest. Extracellular K^+^ levels can specifically regulate the cell-surface expression of Kv11.1 channels, and hypokalemia may enhance Kv11.1 channel internalization and degradation via lysosomal targeting [37], [38]. Hypokalemia would be predicted to further reduce the current density of I_Kr_ channels already impaired at baseline by the P1086fs+32X mutation in our patient, thereby markedly prolonging the QT interval and increasing the risk of torsade arrest in our proband. This may also explain why the proband's sibling was asymptomatic, as he is normokalemic. Another clinical finding of note was the unusual ECG in proband's brother with resting ST elevation along with bifid T waves. Although this phenotype has been described in Brugada syndrome due to Na^+^ channel mutations and gain-of-function Kv11.1 mutant channels [39], it has not been previously reported with dominant-negative Kv11.1 mutations associated with LQT2. Given that both patients were screened for only a subset of genes associated with LQT1-5, it is possible that the brother harbors yet identified mutations or polymorphisms in other proteins.

### Conclusion

We have provided the first clinical description and *in vitro* assessment of the P1086fs+32X LQT2 mutation. This mutation was recently reported in a large clinical database from a multi-center case-control study screening patients for LQT [40]. However, no clinical data or electrophysiological properties of this mutation were provided. The characterization of this unique LQT2 mutant Kv11.1 channel may provide structural information about the Kv11.1 C-terminus, and provide insight related to the process of proteasomal degradation of LQT2 mutants, and the importance of this mechanism in controlling protein function [30], [31]. Clinically, this study stresses the importance of K^+^ supplementation, maintenance and monitoring in LQT2 patients, especially those that may be more susceptible to life-threatening Kv11.1 channel dysfunction associated with severe loss-of-function mutations.
